# Supplementary material for: Disparities in telemedicine use and payment policies in the United States between 2019 and 2023
Source: Commun Med (Lond). 2025 Feb 26;5:52. doi: 10.1038/s43856-025-00757-2 (PMC11865567; doi:10.1038/s43856-025-00757-2)
Supplement: Supplementary file 2 — Supplementary Information [file 43856_2025_757_MOESM2_ESM.docx]

**Supplemental Information**

Supplement for:

**Disparities in telemedicine use and payment policies in the United States between 2019 and 2023**

Anna D. Gage; Megan A. Knight; Corinne Bintz; Robert W. Aldridge; Olivia Angelino; Joseph L. Dieleman; M. Ashworth Dirac; Laura Dwyer-Lindgren; Simon I. Hay; Rafael Lozano; Ali H. Mokdad; Annie Haakenstad

Table of Contents

[Supplementary Table 1. Comparison of analytic sample to the 2019 National Ambulatory Medical Care Survey 1](#_Toc63671063)

[Supplementary Figure 1. Flow diagram of included health systems 2](#_Toc237564656)

[Supplementary Methods 1. Validation of imputation of race and ethnicity 2](#_Toc1700187614)

[Supplementary Table 2. Comparison of patients by race and ethnicity with hold-outs and overall 3](#_Toc227734712)

[Supplementary Figure 2. Telemedicine use by race/ethnicity and imputation 4](#_Toc318939354)

[Supplementary Figure 3. Map of payment parity policies by state 6](#_Toc1941050336)

[Supplementary Methods 2. Regression model for payment parity analysis 6](#_Toc1815691818)

[Supplementary Figure 4. Geographic distribution of the sampled networks 7](#_Toc713332390)

[Supplementary Figure 5. Treatment distribution across units and time 8](#_Toc2133964889)

[Supplementary Figure 6. Association of parity mandates with telemedicine use when treating states that mandated parity after June 2021 as controls 9](#_Toc1020078355)

[Supplementary Figure 7. State-level association of parity mandates on telemedicine use over time 9](#_Toc96263767)

[Supplementary Figure 8. Association of parity mandates with telemedicine use among patients under 65 9](#_Toc112444840)

[Supplementary Table 3. GATHER checklist 10](#_Toc1384330370)

# **Supplementary Table 1. Comparison of analytic sample to the 2019 National Ambulatory Medical Care Survey**

|  | **Healthjump Sample** | **2019 National Ambulatory Medical Care Survey** |
| --- | --- | --- |
| Patient characteristics |  |  |
| Age |  |  |
| Under 15 | 14% | 11% |
| 15-64 | 64% | 53% |
| 65 Plus | 22% | 36% |
| Female | 55% | 58% |
| Race and ethnicity* |  |  |
| White non-Hispanic | 68% | 70% |
| Black non-Hispanic | 15% | 11% |
| Hispanic of any race | 14% | 14% |
| Asian, Native Hawaiian or Other Pacific Islanders | 3% |  |
| American Indian or Alaskan Native | <1% |  |
| Other |  | 5% |

*Race and ethnicity is imputed in the Healthjump sample. Non-imputed data is presented in Supplementary Methods 1.

# **Supplementary Figure 1. Flow diagram of included health systems**


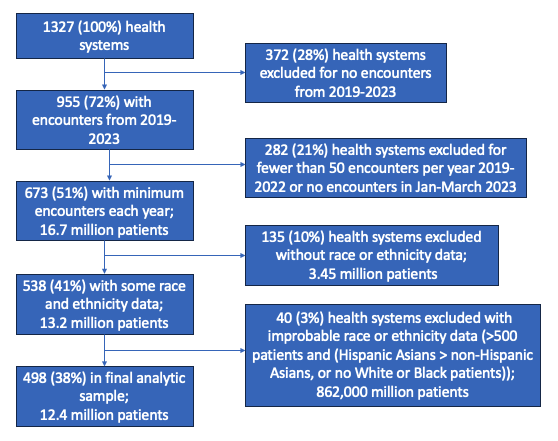


# **Supplementary Methods 1. Validation of imputation of race and ethnicity**

After excluding provider networks that did not provide any data on race or ethnicity or had improbable values for race/ethnicity (i.e. networks with more Hispanic Asian persons than non-Hispanic Asian persons), data on race was missing from 28% of patients and ethnicity from 30% of patients in our sample. We imputed race and ethnicity using multiple imputation and 10 imputations with a logistic regression model for ethnicity and polytomous logistic regression for race using the following covariates: patient age, gender and primary language spoken (English, Spanish, or language of Asian origin), number of patient visits during the study period, the provider network and demographic and economic characteristics of the patient’s 3-digit zip code drawn from the 2010 Census, including percent non-Hispanic white, non-Hispanic Black, Asian, Hispanic, under age 18, over age 65, poverty rate and rural.

We used two primary approaches to assess the validity of our approach to multiple imputation: out-of-sample prediction and comparison with complete case analysis. First, we subset to patients with complete race/ethnicity and created 10 random hold out sets that set race or ethnicity to missing. We then cycled through all the holdout sets and did a single imputation and compared the actual race and ethnicity value to the imputed values. Second, using the full analytic sample, we imputed missing race/ethnicity values ten times using the covariates listed above. We then compared the values of telemedicine use by race and ethnicity for each of imputations, as well as a complete case analysis of unimputed data.

Tables A and B show the comparison of the percent of the population in each race/ethnicity category in the original data versus when race was held out (Table A) and ethnicity was held out (Table B). The greatest misclassification occurs in imputing non-white patients as white non-Hispanic, and vice versa. Table C shows the percent of patients in each category for each imputation and with no imputation. Figures A-E show telemedicine use over the study period for each race and ethnicity group by imputation.

# **Supplementary Table 2. Comparison of patients by race and ethnicity with hold-outs and overall**

**Supplementary Table 2A. Race hold-out**

|  | Imputed |  |  |  |  |  |
| --- | --- | --- | --- | --- | --- | --- |
| Original | AAPI | AI/AN | Black | Hispanic | White | Sum |
| AAPI | 0.3% | 0.0% | 0.4% | 0.0% | 1.9% | 2.6% |
| AI/AN | 0.0% | 0.0% | 0.1% | 0.0% | 0.3% | 0.4% |
| Black | 0.4% | 0.1% | 4.9% | 0.0% | 9.5% | 14.8% |
| Hispanic | 0.0% | 0.0% | 0.0% | 10.5% | 0.0% | 10.5% |
| White | 1.9% | 0.3% | 9.5% | 0.0% | 59.8% | 71.6% |
| Sum | 2.6% | 0.4% | 14.8% | 10.5% | 71.6% | 100.0% |
| Overall accuracy (sum of diagonal) | | | |  |  | 75.5% |

**Supplementary Table 2B. Ethnicity hold-out**

|  | Imputed |  |  |  |  |  |
| --- | --- | --- | --- | --- | --- | --- |
| Original | AAPI | AI/AN | Black | Hispanic | White | **Sum** |
| AAPI | 2.5% | 0.0% | 0.0% | 0.1% | 0.0% | **2.6%** |
| AI/AN | 0.0% | 0.4% | 0.0% | 0.1% | 0.0% | **0.4%** |
| Black | 0.0% | 0.0% | 14.6% | 0.2% | 0.0% | **14.8%** |
| Hispanic | 0.1% | 0.1% | 0.2% | 5.2% | 4.9% | **10.5%** |
| White | 0.0% | 0.0% | 0.0% | 4.9% | 66.7% | **71.6%** |
| Sum | **2.6%** | **0.4%** | **14.8%** | **10.5%** | **71.6%** | **100.0%** |
| Overall accuracy (sum of diagonal) | | | |  |  | **89.4%** |

**Supplementary Table 2C. Patients by race/ethnicity in each imputation**

|  | No impute | 1 | 2 | 3 | 4 | 5 | 6 | 7 | 8 | 9 | 10 |
| --- | --- | --- | --- | --- | --- | --- | --- | --- | --- | --- | --- |
| AAPI | 1.65% | 3.17% | 3.17% | 3.17% | 3.17% | 3.18% | 3.17% | 3.17% | 3.17% | 3.17% | 3.17% |
| AI/AN | 0.25% | 0.40% | 0.40% | 0.41% | 0.40% | 0.40% | 0.40% | 0.40% | 0.40% | 0.40% | 0.40% |
| BNH | 10.07% | 15.25% | 15.25% | 15.25% | 15.25% | 15.25% | 15.25% | 15.26% | 15.25% | 15.26% | 15.25% |
| Hispanic | 8.69% | 13.52% | 13.52% | 13.53% | 13.53% | 13.52% | 13.52% | 13.53% | 13.52% | 13.52% | 13.52% |
| WNH | 43.28% | 67.66% | 67.65% | 67.65% | 67.64% | 67.65% | 67.65% | 67.64% | 67.65% | 67.65% | 67.66% |
| Missing | 36.06% |  |  |  |  |  |  |  |  |  |  |

# **Supplementary Figure 2. Telemedicine use by race/ethnicity and imputation**

**Supplementary Figure 2A. American Indian and Alaskan Native patients**


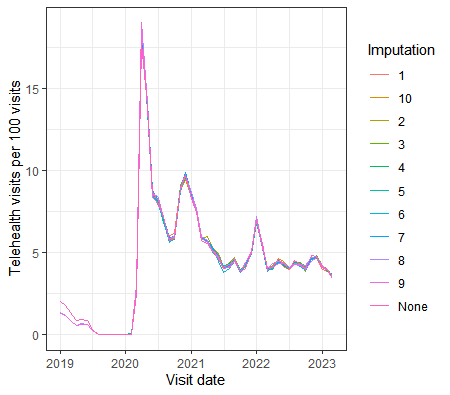


**Supplementary Figure 2B. Asian Americans, Native Hawaiians, and Other Pacific Islander patients**


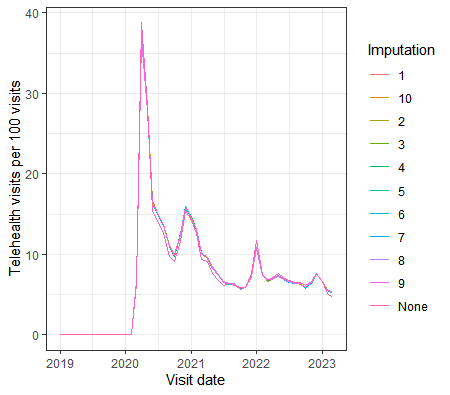


**Supplementary Figure 2C. Black non-Hispanic patients**


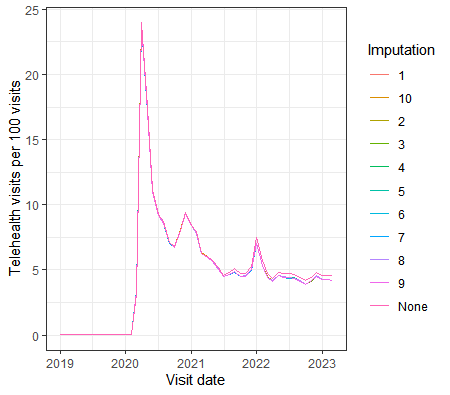


**Supplementary Figure 2D. Hispanic patients**


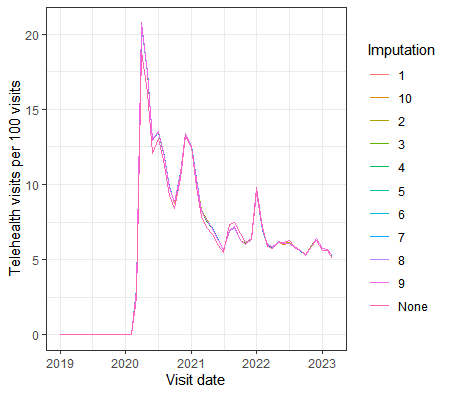


**Supplementary Figure 2E. White non-Hispanic patients**


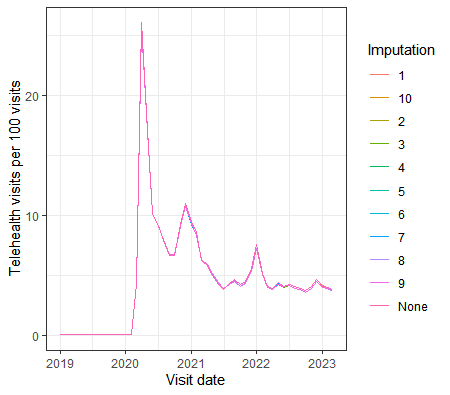


# **Supplementary Figure 3. Map of payment parity policies by state**


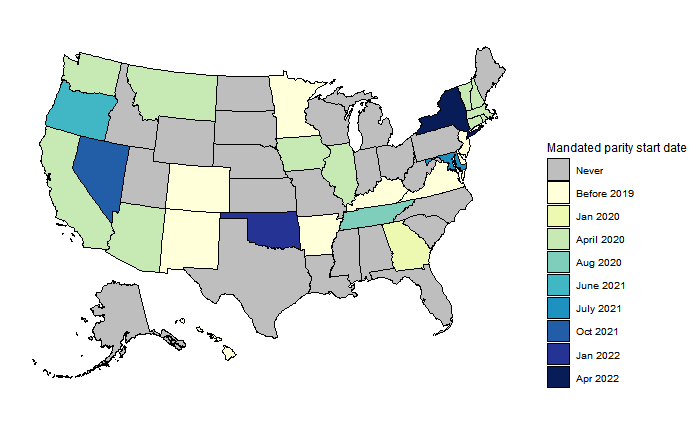


# **Supplementary Methods 2. Regression model for payment parity analysis**

We use the following model to estimate the association between payment parity mandates and telehealth use using the regression model:

$$Y_{ij}=Parity_{i}\cdot\gamma_{j}+ Parity_{i} + \alpha_{i}+ \gamma_{j}+\beta X_{it}+\epsilon_{ij}$$

Where Y is the percent of outpatient consultations conducted via telemedicine for health system *i* in quarter *j,* Parity is a binary variable indicating whether a health system is located in a state that has a payment parity mandate by April 2020, γ is a set of quarter fixed effects from Q1 2019 to Q1 2023, α is a set of health system fixed effects, and X is a set of time-varying health system characteristics: the percent of patients per quarter that were white, Black, Hispanic, under age 18, over age 65; the log number of patients per quarter; the percent change in the patients per quarter from the 2019 average; the mandate propensity index; and percent of days per quarter that the provider network’s state implemented stay at home orders.

The main independent variable of interest is the interaction between Parity and quarter fixed effects, $Parity_{i}\cdot\gamma_{j}$. .

We use the additional models in order to estimate the heterogeneity of the association by health system characteristics:

$Y_{ij}=Parity_{i}*\gamma_{j}*M_{i}+ Parity_{i} + \alpha_{i}+ \gamma_{j}+M_{i}+\beta X_{it}+\epsilon_{ij}$

Where M is category of health system by patient race/ethnicity composition, urbanicity, size and state mandates and the other variables are defined as above.

# **Supplementary Figure 4. Geographic distribution of the sampled networks**


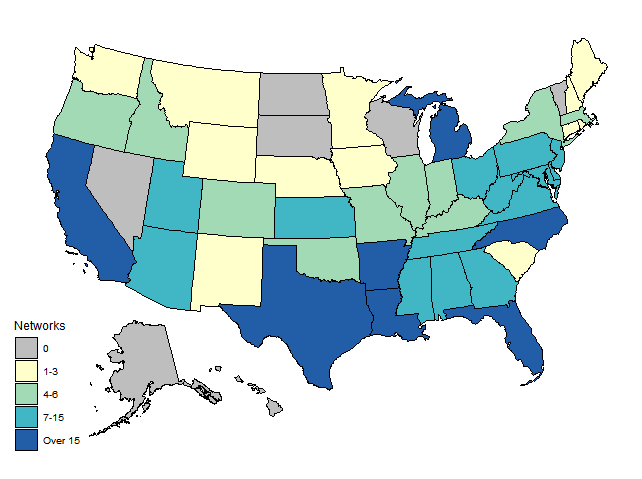


# **Supplementary Figure 5. Treatment distribution across units and time**


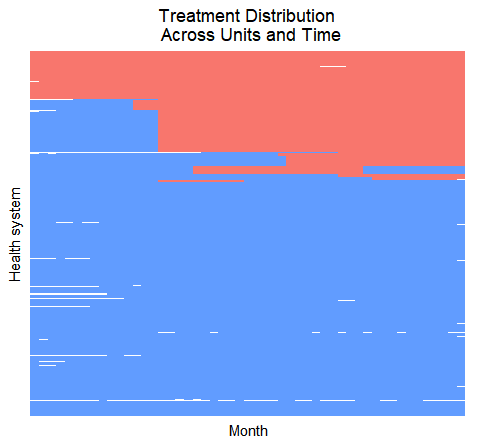


April 2020

March 2023

Jan 2019


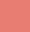

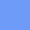


State mandated payment parity

State did not mandate payment parity

# **Supplementary Figure 6. Association of parity mandates with telemedicine use when treating states that mandated parity after June 2021 as controls**


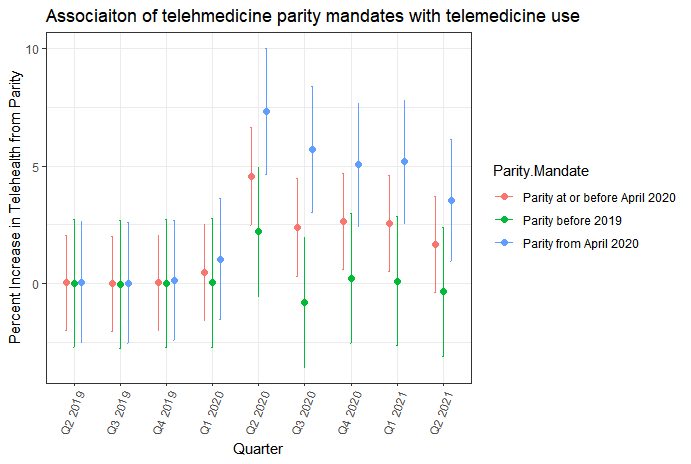


# **Supplementary Figure 7. State-level association of parity mandates on telemedicine use over time**


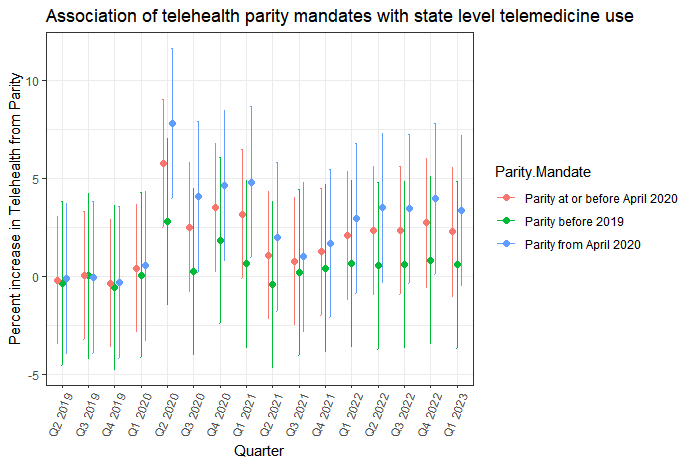


# **Supplementary Figure 8. Association of parity mandates with telemedicine use among patients under 65**


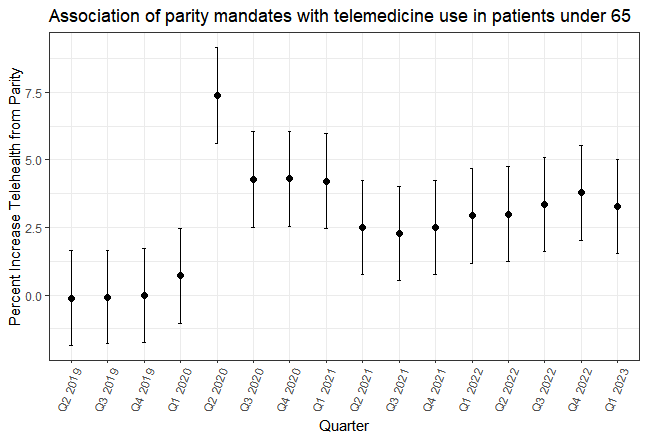


# **Supplementary Table 3. GATHER checklist**

| **Item #** | **GATHER checklist item** | **Description of compliance** | **Reference** |
| --- | --- | --- | --- |
| **Objectives and funding** | | | |
| 1 | Define the indicators, populations, and time periods for which estimates were made. | Description of indicators, definitions, relevant time periods, and populations in paper. | Data and participants section of main text, and Variable definitions section of main text |
| 2 | List the funding sources for the work. | Funding sources listed in paper. | Acknowledgements section of main text |
| **Data Inputs** | | | |
| *For all data inputs from multiple sources that are synthesized as a part of the study:* | | | |
| 3 | Describe how the data were identified and how the data were accessed. | Description of accessing data is described in paper. | Data availability section of main text, and Acknowledgments section of main text |
| 4 | Specify the inclusion and exclusion criteria. Identify all ad-hoc exclusions. | Description of inclusion and exclusion criteria noted in paper and supplementary information. | Data and participants section of main text, and Supplementary Figure 1 |
| 5 | Provide information on all included data sources and their main characteristics. For each data source used, report reference information or contact name/institution, population represented, data collection method, year(s) of data collection, sex and age range, diagnostic criteria or measurement method, and sample size, as relevant. | Description of data sources are included in paper and supplementary information. | Data and participants section of main text, Table 1, Data availability section of main text, and Supplementary Table 1 |
| 6 | Identify and describe any categories of input data that have potentially important biases (eg, based on characteristics listed in item 5). | Summary of some known biases included in paper. | Data and participants section of main text, and Discussion section of main text |
| *For data inputs that contribute to the analysis but were not synthesized as part of the study:* | | | |
| 7 | Describe and give sources for any other data inputs. | N/A | N/A |
| *For all data inputs:* | | | |
| 8 | Provide all data inputs in a file format from which data can be efficiently extracted (eg, a spreadsheet as opposed to a PDF), including all relevant meta-data listed in item 5. For any data inputs that cannot be shared due to ethical or legal reasons, such as third-party ownership, provide a contact name or the name of the institution that retains the right to the data. | Description of institution that retains data rights is included in paper. | Data availability section of main text |
| **Data Analysis** | | | |
| 9 | Provide a conceptual overview of the data analysis method. A diagram may be helpful. | Written overview of data analysis method is included in paper. | Statistics and reproducibility section of main text |
| 10 | Provide a detailed description of all steps of the analysis, including mathematical formulae. This description should cover, as relevant, data cleaning, data pre-processing, data adjustments and weighting of data sources, and mathematical or statistical model(s). | Detailed descriptions of data analysis methods are included in paper and supplementary information. | Variable definitions section of main text, Statistics and reproducibility section of main text, and Supplementary Methods 1-2 |
| 11 | Describe how candidate models were evaluated and how the final model(s) were selected. |  | Models evaluated against the different specifications outlined in the robustness checks |
| 12 | Provide the results of an evaluation of model performance, if done, as well as the results of any relevant sensitivity analysis. | Provided the results of an evaluation of model performance. | Results section of main text, and Supplementary methods 1 |
| 13 | Describe methods for calculating uncertainty of the estimates. State which sources of uncertainty were, and were not, accounted for in the uncertainty analysis. |  | Variable definitions section of the main text, and Statistics and reproducibility section of the main text. |
| 14 | State how analytic or statistical source code used to generate estimates can be accessed. | Availability of analytic source code is discussed paper and posted in publicly available GitHub repository. | Code availability section of main text |
| **Results and discussion** | | | |
| 15 | Provide published estimates in a file format from which data can be efficiently extracted. | Data underlying main figures are provided in paper. | Data availability section of main text, and Supplementary Data 3 |
| 16 | Report a quantitative measure of the uncertainty of the estimates (eg, uncertainty intervals). | Uncertainty intervals are provided with all estimated results. | Results section of main text, Figure 1, Figure 4, and Supplementary Figures 6-8 |
| 17 | Interpret results in light of existing evidence. If updating a previous set of estimates, describe the reasons for changes in estimates. | Contextualizing results in existing evidence provided in the paper. | Introduction section of main text, and Discussion section of main text |
| 18 | Discuss limitations of the estimates. Include a discussion of any modelling assumptions or data limitations that affect interpretation of the estimates. | Discussion of limitations provided in the narrative of the paper. | Discussion section of main text |
