# Supplementary material for: Disparities in telemedicine use and payment policies in the United States between 2019 and 2023
Source: Commun Med (Lond). 2025 Feb 26;5:52. doi: 10.1038/s43856-025-00757-2 (PMC11865567; doi:10.1038/s43856-025-00757-2)
Supplement: Supplementary file 3 — Description of Additional Supplementary Files [file 43856_2025_757_MOESM3_ESM.pdf]

Description of Additional Supplementary Files

File name- Supplementary Data 1

File description- Payment policies by State

File name- Supplementary Data 2

File description- Association of telemedicine payment parity policies with telemedicine use by system characteristic

File name- Supplementary Data 3

File description- Data underlying main figures
