# Supplementary material for: Disparities in telemedicine use and payment policies in the United States between 2019 and 2023
Source: Commun Med (Lond). 2025 Feb 26;5:52. doi: 10.1038/s43856-025-00757-2 (PMC11865567; doi:10.1038/s43856-025-00757-2)
Supplement: Supplementary file 7 — Reporting Summary [file 43856_2025_757_MOESM7_ESM.pdf]

nature portfolio

Corresponding author(s): Annie Haakenstad

Last updated by author(s): May 23, 2024

Reporting Summary

Nature Portfolio wishes to improve the reproducibility of the work that we publish. This form provides structure for consistency and transparency in reporting. For further information on Nature Portfolio policies, see our Editorial Policies and the Editorial Policy Checklist.

Statistics

For all statistical analyses, confirm that the following items are present in the figure legend, table legend, main text, or Methods section.

n/a	Confirmed	
☐	☒	The exact sample size (*n*) for each experimental group/condition, given as a discrete number and unit of measurement
☐	☒	A statement on whether measurements were taken from distinct samples or whether the same sample was measured repeatedly
☐	☒	The statistical test(s) used AND whether they are one- or two-sided<br>*Only common tests should be described solely by name; describe more complex techniques in the Methods section.*
☐	☒	A description of all covariates tested
☐	☒	A description of any assumptions or corrections, such as tests of normality and adjustment for multiple comparisons
☐	☒	A full description of the statistical parameters including central tendency (e.g. means) or other basic estimates (e.g. regression coefficient) AND variation (e.g. standard deviation) or associated estimates of uncertainty (e.g. confidence intervals)
☐	☒	For null hypothesis testing, the test statistic (e.g. *F*, *t*, *r*) with confidence intervals, effect sizes, degrees of freedom and *P* value noted<br>*Give P values as exact values whenever suitable.*
☒	☐	For Bayesian analysis, information on the choice of priors and Markov chain Monte Carlo settings
☒	☐	For hierarchical and complex designs, identification of the appropriate level for tests and full reporting of outcomes
☐	☒	Estimates of effect sizes (e.g. Cohen's *d*, Pearson's *r*), indicating how they were calculated

Our web collection on statistics for biologists contains articles on many of the points above.

Software and code

Policy information about availability of computer code

Data collection	No software was used for data collection.
Data analysis	Analyses were using R version 4.0.5. All code used for these analyses is publicly available online (https://github.com/ihmeuw/us_telemedicine).

For manuscripts utilizing custom algorithms or software that are central to the research but not yet described in published literature, software must be made available to editors and reviewers. We strongly encourage code deposition in a community repository (e.g. GitHub). See the Nature Portfolio guidelines for submitting code & software for further information.

Data

Policy information about availability of data

All manuscripts must include a data availability statement. This statement should provide the following information, where applicable:

- Accession codes, unique identifiers, or web links for publicly available datasets
- A description of any restrictions on data availability
- For clinical datasets or third party data, please ensure that the statement adheres to our policy

The secondary data used for this study are Electronic Health Record data including diagnosis, procedures and histories sourced from participating members of the Healthjump network. Data are not available publicly, but can be accessed via application to the COVID-19 Research Database, www.covid19researchdatabase.org.

Human research participants

Policy information about studies involving human research participants and Sex and Gender in Research.

Reporting on sex and gender	Distribution of sex of research participants is included in Table 1. Sex and gender is otherwise not a focus of this study.
Population characteristics	Outlined in Table 1
Recruitment	This study using secondary data did not participate in any recruitment of study participants. Study uses EHR data from patients who received healthcare from participating members of the Healthjump network. The data that was provided to researchers was completely de-identified.
Ethics oversight	University of Washington

Note that full information on the approval of the study protocol must also be provided in the manuscript.

Field-specific reporting

Please select the one below that is the best fit for your research. If you are not sure, read the appropriate sections before making your selection.

☐ Life sciences ☒ Behavioural & social sciences ☐ Ecological, evolutionary & environmental sciences

For a reference copy of the document with all sections, see nature.com/documents/nr-reporting-summary-flat.pdf

Behavioural & social sciences study design

All studies must disclose on these points even when the disclosure is negative.

Study description	Quantitative cross-sectional
Research sample	United States healthcare providers participating in the Healthjump network.
Sampling strategy	Convenience sample of healthcare systems that opted to participate in the Healthjump network. Within the group of participating systems, we included all systems that met our inclusion criteria stated below.
Data collection	Data were collected via EHR as part of routine healthcare provision. The de-identified data were then provided to the researchers via the Covid-19 Research Database.
Timing	We included EHR records from January 1, 2019 to March 31, 2023.
Data exclusions	To limit the impact of new units joining or leaving the database, we included only systems that report at least 50 consultations per year during 2019–2022 and at least one consultation during the first three months of 2023. We further excluded health systems that did not report any data on patient race or ethnicity or had improbable race or ethnicity values. We additionally excluded inpatient and emergency room visits and patient records missing three-digit ZIP code, gender, or birth year information (<0.1% of patients). Exclusions are depicted in Supplementary Figure 1.
Non-participation	282 networks were excluded because they did not include the minimum number of consultations during the entire duration of the study period (e.g. they joined or left the Healthjump network during the study period).
Randomization	Participants were not allocated into experimental groups.

Reporting for specific materials, systems and methods

We require information from authors about some types of materials, experimental systems and methods used in many studies. Here, indicate whether each material, system or method listed is relevant to your study. If you are not sure if a list item applies to your research, read the appropriate section before selecting a response.

Materials & experimental systems

n/a	Involved in the study
☒	☐ Antibodies
☒	☐ Eukaryotic cell lines
☒	☐ Palaeontology and archaeology
☒	☐ Animals and other organisms
☒	☐ Clinical data
☒	☐ Dual use research of concern

Methods

n/a	Involved in the study
☒	☐ ChIP-seq
☒	☐ Flow cytometry
☒	☐ MRI-based neuroimaging
